# Chemical insights into the roles of nanowire cores on the growth and supercapacitor performances of Ni-Co-O/Ni(OH)_2_ core/shell electrodes

**DOI:** 10.1038/srep21566

**Published:** 2016-02-09

**Authors:** Xuesong Yin, Chunhua Tang, Liuyang Zhang, Zhi Gen Yu, Hao Gong

**Affiliations:** 1 Department of Materials Science and Engineering, National University of Singapore, 117576, Singapore; 2 Institute of High Performance Computing, 1 Fusionopolis Way, Singapore 138632, Singapore

## Abstract

Nanostructured core/shell electrodes have been experimentally demonstrated promising for high-performance electrochemical energy storage devices. However, chemical insights into the significant roles of nanowire cores on the growth of shells and their supercapacitor behaviors still remain as a research shortfall. In this work, by substituting 1/3 cobalt in the Co_3_O_4_ nanowire core with nickel, a 61% enhancement of the specific mass-loading of the Ni(OH)_2_ shell, a tremendous 93% increase of the volumetric capacitance and a superior cyclability were achieved in a novel NiCo_2_O_4_/Ni(OH)_2_ core/shell electrode in contrast to a Co_3_O_4_/Ni(OH)_2_ one. A comparative study suggested that not only the growth of Ni(OH)_2_ shells but also the contribution of cores were attributed to the overall performances. Importantly, their chemical origins were revealed through a theoretical simulation of the core/shell interfacial energy changes. Besides, asymmetric supercapacitor devices and applications were also explored. The scientific clues and practical potentials obtained in this work are helpful for the design and analysis of alternative core/shell electrode materials.

Electric energy storage devices with both large energy capacity (energy density) and fast charging ability (power density) are highly demanded nowadays in numerous applications^1^,^2^,^3^. Traditional capacitors principally employ fast static electric storage but exhibit relatively low energy density, and batteries normally involve slow electrode reactions to realize relatively high energy densities^4^,^5^. As a bridge between capacitors and batteries, electrochemical capacitors, also known as supercapacitors, have shown great potentials to balance energy density and power density by combining fast surface-tended electrochemical reactions and static electric storage^6^,^7^. Currently, most commercial supercapacitors utilize carbon-based materials, due to their large specific areas, low costs and compatibility with various electrolytes^8^,^9^,^10^. Categorized as electrochemical double layer capacitors (EDLCs), the specific capacitances of carbon based supercapacitors are limited by their less electrochemically active nature^8^,^11^. To achieve higher capacitances, electrochemically active electrode materials are developed for supercapacitors, known as pseudo-capacitors^6^,^7^,^12^. Nano-structured transition metal oxides/hydroxides, such as RuO_x_, MnO_2_, MoO_x_, CoO_x_/(OH)_x_ and NiO_x_/(OH)_x_, become very attractive candidates due to their multiple electronic states and flexibility of morphological manipulation^13^,^14^,^15^,^16^,^17^,^18^,^19^,^20^,^21^,^22^,^23^. Among them, Ni(OH)_2_ is one of the most outstanding candidates because of its high gravimetric capacitance, low-cost, non-toxicity and easy accessibility to nanostructures^20^,^24^,^25^.

Although experimental gravimetric capacitances of Ni(OH)_2_ close to its theoretical value were achieved in literature, the small loadings limit their applications as real capacitor devices^26^,^27^. Especially, the difficulty in growing high loading of active material on substrates leads to a small total capacitance in a unit volume or areal, which reduces the practical significance of these electrodes to a great extent^28^. Practically, 3D Ni foams (NF) have been applied as the substrate due to their relatively high surface area, conductivity and corrosion resistance to alkaline electrolyte for Ni(OH)_2_ based supercapacitor. Despite its much enlarged surface area than a flat substrate, there is still a very high percentage of unused pore space within the Ni foam attributed to its relatively large pore size. Therefore nanowire cores were grown on nickel foams prior to the growth of active material to form core/shell structure that extends into the previously unused pore space. This approach can make more pore space usable in NF and the loading of active material can be dramatically increased as the available surface area is greatly enlarged for the deposition of active materials. Various materials, such as carbon-based nanomaterials^29^,^30^,^31^,^32^,^33^,^34^, TiN^35^, TiO_2_^36^, FeO_x_^37^, NiS_x_^38^, Ni^39^, (Ni,Co)O_x_/(OH)_x_^39^,^40^,^41^, have been employed as cores for Ni(OH)_2_ shell growth. The Ni, Co oxides based cores are of great interests, because they not just serve as an agent to increase the surface area but also contribute to the total capacitance owing to their own high electrochemical activities^40^,^42^,^43^,^44^,^45^,^46^,^47^,^48^,^49^. Tang *et al*. reported a Co_3_O_4_ nanowires/Ni(OH)_2_ core/shell hybrid on nickel foam electrode and achieved a specific capacitance around 15 Fcm^−2^ at a current of 5 mAcm^−2^ 41. NiCo_2_O_4_ nanowires have shown better supercapacitor performance than Co_3_O_4_. However, NiCo_2_O_4_ nanowires/Ni(OH)_2_ core/shell formation and its supercapacitor performance are unclear. It is interesting to find out whether the supercapacitor performance can be increased if the nanowire core is changed to NiCo_2_O_4_ as the substrate for the growth of the same Ni(OH)_2_ shell. It is more interesting to find whether a partial substitution of Co by Ni in the core can significantly affect the growth and performance of Ni(OH)_2_ shell. A comparative study on the Ni(OH)_2_ shell growing on Co_3_O_4_ and NiCo_2_O_4_ nanowire core materials and their performances in supercapactor devices are worth to be conducted.

In this work, we carried out a comparative study on supercapacitor performances for the core/shell structures of Ni(OH)_2_ grown on two different nanowires: NiCo_2_O_4_ and Co_3_O_4_. It was found that NiCo_2_O_4_/Ni(OH)_2_ core/shell structure interestingly showed a great improvement in supercapacitor performance comparing with the Co_3_O_4_/Ni(OH)_2_ core/shell electrode. The volumetric and gravimetric capacitances increased 93% and 56%, respectively. And the capacitance retention also enhanced to 96.5% for the NiCo_2_O_4_/Ni(OH)_2_ electrode compared with the Co_3_O_4_/Ni(OH)_2_ (74.4%) after 1000 cycles. Both the enhancement of the specific mass-loading of the Ni(OH)_2_ shell and the more electrochemically active NiCo_2_O_4_ core contributed to its superior performances. Through theoretical simulations, the chemical adsorption energy between the NiCo_2_O_4_ core and Ni(OH)_2_ shell was found to be smaller than that of the Co_3_O_4_/Ni(OH)_2_ structure, which revealed the mechanisms behind the influences of such a compositional change in the nanowire core material on the core/shell’s properties. In addition, asymmetric supercapacitor devices were fabricated to demonstrate their great potentials for practical applications. The experimental evidences and scientific understandings achieved in this work are of great values for the design and interpretation of other core/shell systems.

## Results and Discussion

The core/shell electrode preparation involves a hydrothermal deposition of Ni-Co-O nanowire cores on nickel foam (NF) and a chemical bath deposition of Ni(OH)_2_ shells on the nanowires as illustrated in Fig. S1.

**Figure 1:**
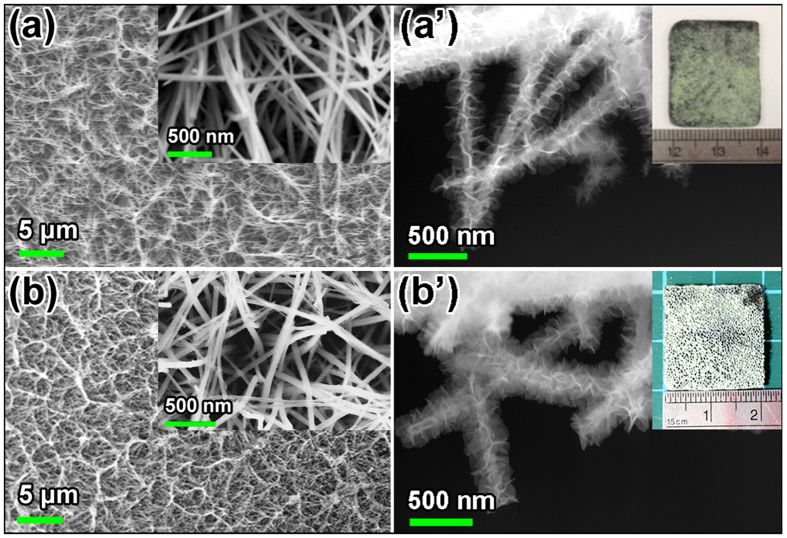
SEM images of (**a**) Co_3_O_4_ and (**b**) NiCo_2_O_4_ nanowires with magnified images in their insets, and (**a**’) Co_3_O_4_/Ni(OH)_2_ and (**b**’) NiCo_2_O_4_/Ni(OH)_2_ core/shell structures on NF with their photos in the insets.

SEM images of the cores, Co_3_O_4_ and NiCo_2_O_4_ nanowires, and the core/shell structures, Co_3_O_4_/Ni(OH)_2_ and NiCo_2_O_4_/Ni(OH)_2_, are shown in Fig. 1. Uniform coverages of Co_3_O_4_ and NiCo_2_O_4_ nanowires on NFs are observed in Fig. 1a,b. Their insets provide the magnified images of these nanowires. It is clear that the Co_3_O_4_ and NiCo_2_O_4_ nanowires are well assembled with 3D network architectures, which are favorable to provide large surface areas for further active material growth. The mass-loadings for the Co_3_O_4_ and NiCo_2_O_4_ nanowires are 19.3 mg and 20.9 mg as shown in Table 1(I). After chemical bath deposition of Ni(OH)_2_, both the NF/Co_3_O_4_/Ni(OH)_2_ and NF/NiCo_2_O_4_/Ni(OH)_2_ electrodes turn to be light green as shown in the insets of Fig. 1a’. Figure 1a’ and their insets demonstrate a good coverage and uniform growth of nanostructured gauze-like Ni(OH)_2_ shells wrapping the Co_3_O_4_ and NiCo_2_O_4_ nanowire cores, but a more complete coverage appears at the sample with NiCo_2_O_4_ nanowire cores. And the mass-loadings of Ni(OH)2 shell on the Co_3_O_4_ and NiCo_2_O_4_ nanowires are 28.3 mg and 37.7 mg as seen in Table 1(I).

**Figure 2:**
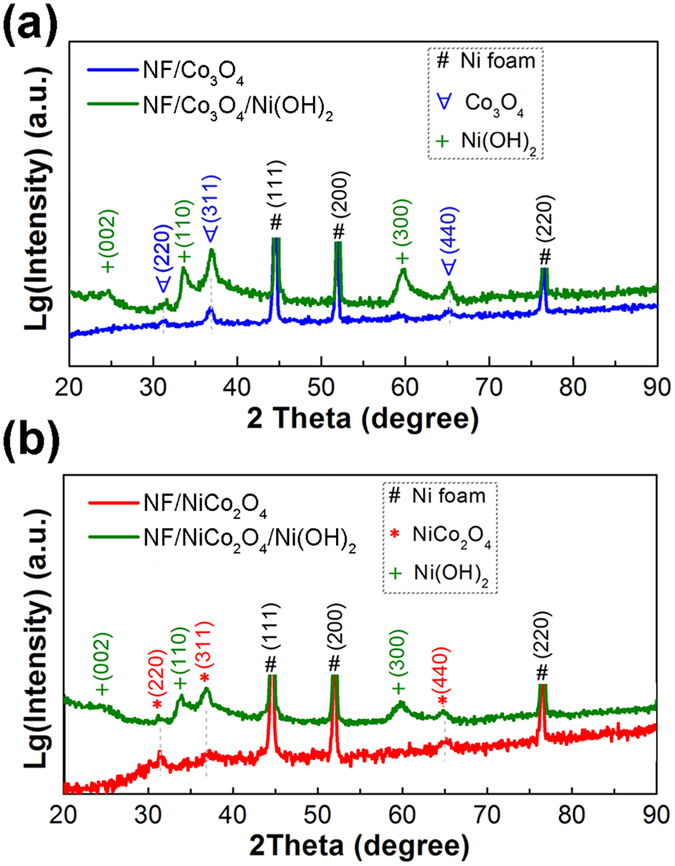
XRD patterns of (**a**) NF/Co_3_O_4_ and NF/Co_3_O_4_/Ni(OH)_2_, and (**b**) NF/NiCo_2_O_4_ and NF/NiCo_2_O_4_/Ni(OH)_2_ samples.

XRD patterns of the NF/Co_3_O_4_ and NF/Co_3_O_4_/Ni(OH)_2_ samples and the NF/NiCo_2_O_4_ and NF/NiCo_2_O_4_/Ni(OH)_2_ samples are presented in Fig. 2a,b, respectively. The peaks at 44.4°, 51.6° and 76.1° in all of these samples are assigned to (111), (200) and (220) planes of the metallic Ni phase (JCPDS #01-1258). In Fig. 2a, the peaks at 31.3°, 36.9° and 44.8° are indexed to (220), (311) and (440) planes of the Co_3_O_4_ phase (JCPDS #42-1467). The peaks at 31.1°, 36.7° and 44.6° can be indexed to (220), (311) and (440) planes of NiCo_2_O_4_ (JCPDS #20-0781) in Fig. 2b. In the XRD patterns of the NF/Co_3_O_4_/Ni(OH)_2_ and NF/NiCo_2_O_4_/Ni(OH)_2_ electrodes, in addition to the peaks referring to the NF/Co_3_O_4_ and NF/NiCo_2_O_4_ cores, the diffraction peaks at 23.8°, 33.7° and 59.6° belongs to (002), (110) and (300) planes of *α*-Ni(OH)_2_ phase (JCPDS #22-0444).

**Figure 3:**
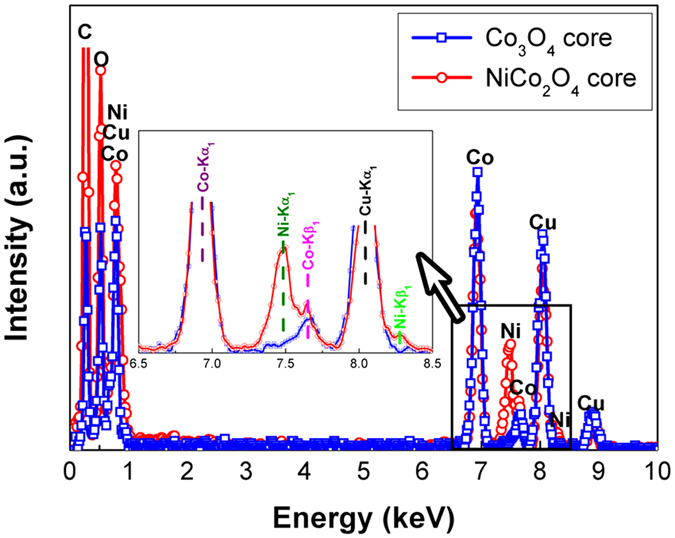
EDS profiles of Co_3_O_4_ and NiCo_2_O_4_ nanowires, and the signals in the energy range from 6.5 keV to 8.5 keV are magnified in the inset.

EDS results for Co_3_O_4_ and NiCo_2_O_4_ nanowires are obtained using the transmission electron microscope equipped with EDS facility as shown in Fig. 3. To distinguish Ni and Co, the profiles in the energy region between 6.5 keV and 8 keV are magnified in the inset. For the Co_3_O_4_ nanowire, only Co *K*_α1_ (6.931 keV) and Co *K*_β1_ (7.649 keV) peaks are detected. For the NiCo_2_O_4_ nanowire, additional Ni *K*_α1_ (7.480 keV) and Ni *K*_β1_ (8.267 keV) peaks appear. The EDS spectra data analysis reveals a Ni/Co ratio close to the formulated ratio for NiCo_2_O_4_, but this ratio cannot be accurate due to the overlapping of Ni *K*_α1_ and Co *K*_β1_ peaks. The Cu and C signals come from the TEM grid.

**Figure 4:**
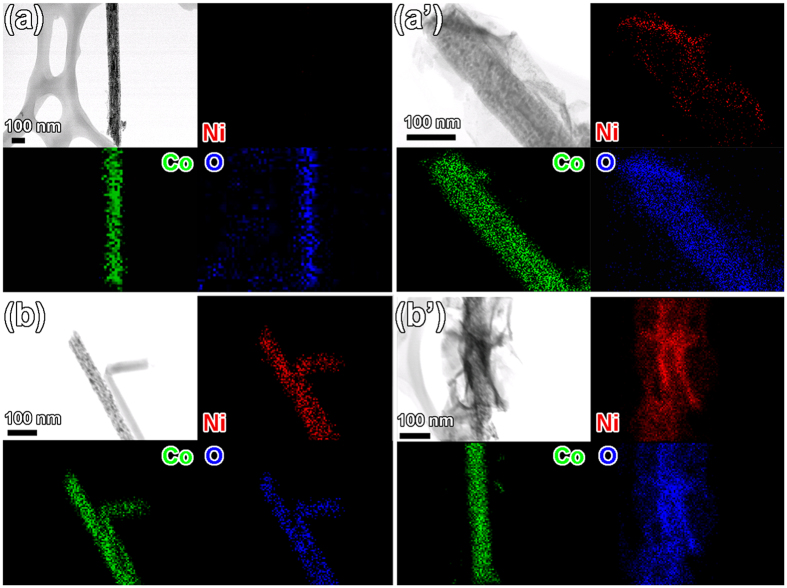
STEM and elemental mapping images of (**a**) Co_3_O_4_ and (**b**) NiCo_2_O_4_ nanowires, and (**a**’) Co_3_O_4_/Ni(OH)_2_ and (**b**’) NiCo_2_O_4_/Ni(OH)_2_ core/shell structures, respectively.

Individual core and core/shell wires are inspected by using scanning transmission electron microscopy (STEM) and elemental mapping. The STEM and elemental mapping images in Fig. 4 show (a) evenly distributed Co and O elements in the Co_3_O_4_ nanowire core and (b) Ni, Co and O elements in the NiCo_2_O_4_ core. For Co_3_O_4_/Ni(OH)_2_ core/shell (Fig. 4a’) and NiCo_2_O_4_/Ni(OH)_2_ core/shell (Fig. 4b’), the STEM and elemental mapping images reveal core/shell structures with the nanowire cores surrounded by the Ni(OH)_2_ flake shell. It can be found that more Ni(OH)_2_ flakes are on the NiCo_2_O_4_ nanowire, in comparison to the Ni(OH)_2_ on the Co_3_O_4_ nanowire, which is in consistent with the SEM observation. The reasons of more Ni(OH)_2_ grown on the NiCo_2_O_4_ nanowire will be discussed later.

**Figure 5:**
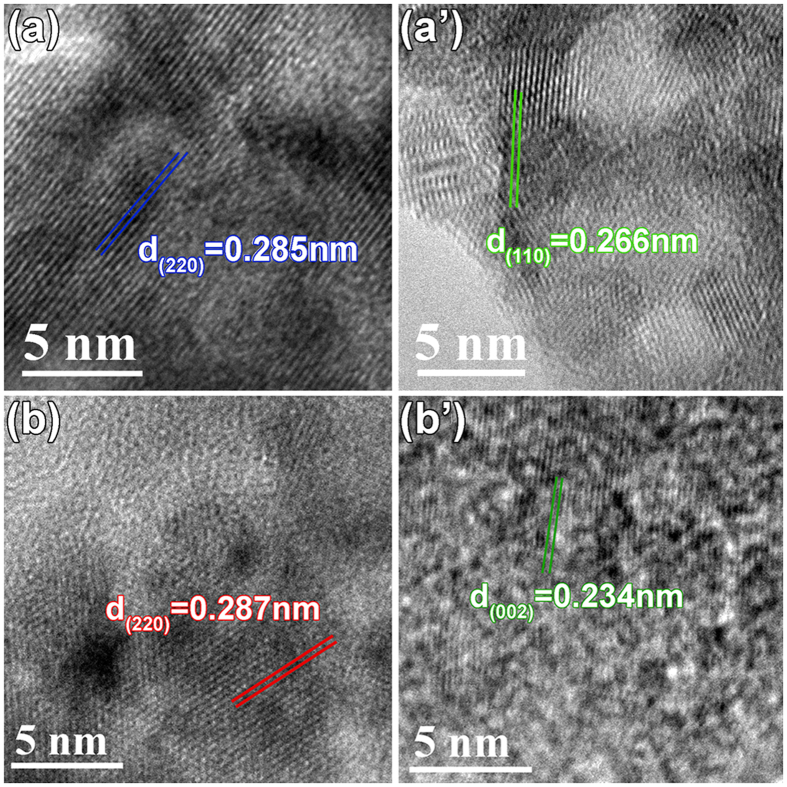
High resolution TEM images of (**a**) Co_3_O_4_ and (**b**) NiCo_2_O_4_ cores, and Ni(OH)_2_ shells on (**a**’) Co_3_O_4_ and (**b**) NiCo_2_O_4_ core, respectively.

The high resolution TEM images of the Co_3_O_4_ and NiCo_2_O_4_ cores (Fig. 5a,b) show clear lattice fringes with spacing values of 0.285 nm for the (220) plane of Co_3_O_4_ and 0.287 nm for the (220) plane of NiCo_2_O_4_, respectively. For the flakes, lattice fringes with spacing values of 0.266 nm and 0.231 nm as indicated in Fig. 5a’ correspond to the (110) and (200) planes of the *α*-Ni(OH)_2_ phase (JCPDS #22-0444).

Herein, it can be concluded that the core/shell structures consisting of the Co_3_O_4_ or NiCo_2_O_4_ nanowire core and the flaky Ni(OH)_2_ shell are successfully deposited on the nickel foam substrate. Counted on their favorable nanostructures of large surface areas and good coverages over the substrates with considerable loadings, it is desired to have good electrochemical performances for these core/shell electrodes in supercapacitor devices.

**Figure 6:**
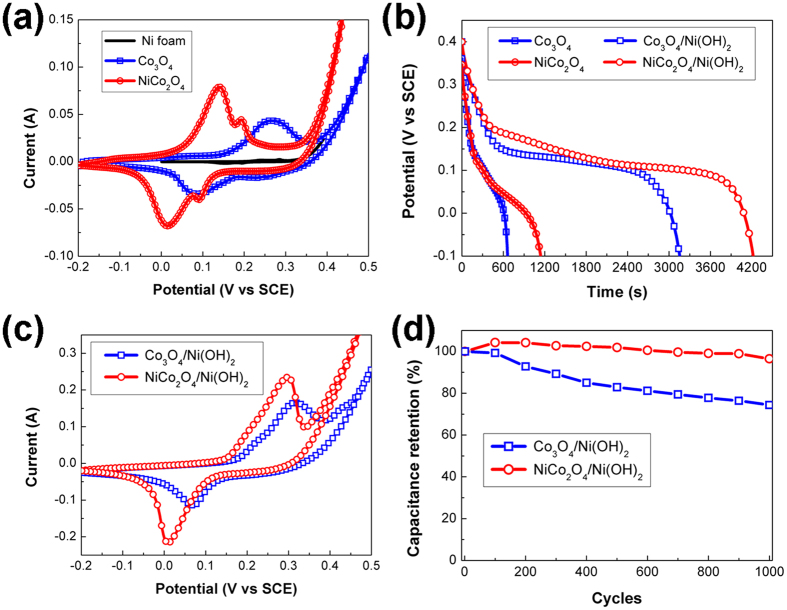
CV curves of (**a**) bare NF, NF/Co_3_O_4_ and NF/NiCo_2_O_4_ electrodes and (**c**) NF/Co_3_O_4_/Ni(OH)_2_ and NF/NiCo_2_O_4_/Ni(OH)_2_ electrodes at a scan rate of 1 mVs^−1^, (**b**) Galvanostatic discharge curves of NF/Co_3_O_4_, NF/NiCo_2_O_4_, NF/Co_3_O_4_/Ni(OH)_2_ and NF/NiCo_2_O_4_/Ni(OH)_2_ electrodes at a discharge current density of 2.5 mA/cm^2^, respectively, (**b**) Capacitance retention of the NF/Co_3_O_4_/Ni(OH)_2_ and NF/NiCo_2_O_4_/Ni(OH)_2_ electrodes at a charge-discharge current of 50 mA/cm^2^ for 1000 cycles.

The capacitive performances of the NF/Co_3_O_4_, NF/NiCo_2_O_4_, NF/Co_3_O_4_/Ni(OH)_2_ and NF/NiCo_2_O_4_/Ni(OH)_2_ electrodes were characterized and shown in Fig. 6. CV curves of NF/Co_3_O_4_ and NF/NiCo_2_O_4_ are shown in Fig. 6a. One pair of redox reaction peaks at 0.08 V and 0.26 V for the NF/Co_3_O_4_ electrode suggests cobalt ion states transformation during the potential sweep^50^,^51^. Distinct two pairs of redox reactions are observed at 0.14 V, 0.02 V and 0.19 V, 0.09 V in the NF/NiCo_2_O_4_ electrode, which is similar to the reported features of NiCo_2_O_4_ based electrodes^52^,^53^. In addition, the CV curve of a bare NF presents a negligible contribution compared with the nanowire core coated electrodes in Fig. 6a. The enclosed area of the CV loop for NF/NiCo_2_O_4_ is also larger than NF/Co_3_O_4_, indicating a larger capacitance value of NF/NiCo_2_O_4_ than NF/Co_3_O_4_. This is consistent with the longer discharging time in the discharge curve of NF/NiCo_2_O_4_ than NF/Co_3_O_4_ in Fig. 6b. The calculated gravimetric, volumetric and areal specific capacitances of core and core/shell electrodes at a current density of 2.5 mA/cm^2^ are presented in Table 1(II). The current density dependent discharge and specific capacitance curves for those core and core/shell electrodes are also presented in Figs S2 and S3. It should be pointed out that in this work the specific capacitance (SC) values were calculated from the discharge measurements, following Equation (1),
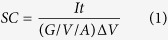
where *I* and *t* are the discharge current and time and Δ*V* (V) is the potential window. *G*, *V* or *A* is the mass, volume or area of the electrode, referring to specific gravimetric (*G*), volumetric (*V*) or areal (*A*) capacitance, respectively. The gravimetric values are based on the active materials.

Table 1(II) shows a 43% higher gravimetric capacitance for NF/NiCo_2_O_4_ than NF/Co_3_O_4_. The volumetric and areal capacitances for NF/NiCo_2_O_4_ are 55% higher than those for NF/Co_3_O_4_. After Ni(OH)_2_ loading, the discharge time for both the NF/Co_3_O_4_/Ni(OH)_2_ and NF/NiCo_2_O_4_/Ni(OH)_2_ electrodes are much prolonged comparing with their respective NF/core samples in Fig. 6b, which reveals much increased capacitances attributed to the Ni(OH)_2_ shell for both core/shell electrodes. The gravimetric specific capacitance for NF/NiCo_2_O_4_/Ni(OH)_2_ is 2079 F/g, which is 68% higher than that of NF/NiCo_2_O_4_. The gravimetric specific capacitance for NF/Co_3_O_4_/Ni(OH)_2_ is 1330 F/g, 54% higher than that of NF/Co_3_O_4_. Furthermore, the longer discharging time of NF/NiCo_2_O_4_/Ni(OH)_2_ than NF/Co_3_O_4_/Ni(OH)_2_ also expresses a higher capacitance of NF/NiCo_2_O_4_/Ni(OH)_2_ when comparing with NF/Co_3_O_4_/Ni(OH)_2_. Moreover, the gravimetric and volumetric specific capacitances of NF/NiCo_2_O_4_/Ni(OH)_2_ contrasting to NF/Co_3_O_4_/Ni(OH)_2_ are increased about 56% and 93%, which are much larger than the relative loading increase of Ni(OH)_2_ (36%). Therefore, it can be concluded that the increased loading of Ni(OH)_2_ shell results in a crucial capacitance enhancement, but the capacitances of the core/shell electrodes are not fully contributed by the Ni(OH)_2_ shell but also counted on the cores’ contribution. Additional evidences are provided by the CV curves of the NF/Co_3_O_4_/Ni(OH)_2_ and NF/NiCo_2_O_4_/Ni(OH)_2_ electrodes in Fig. 6c. After the growth of Ni(OH)_2_ shell, the enclosed areas of CV loops for both core/shell electrodes are significantly extended in contrast to their core electrodes (Fig. 6a). Similar phenomena were also observed in other Ni/Co oxides or hydroxides based core/shell electrodes^12^,^39^,^53^,^54^,^55^. Comparing with NF/Co_3_O_4_/Ni(OH)_2_, there are traceable characteristics of left-shifted electrochemical reaction peaks in NF/NiCo_2_O_4_/Ni(OH)_2_, which could be resulted from the similar features in the CV loops of their cores in Fig. 6a. In Fig. 6d, the cycling performances of these two core/shell electrodes are examined at a charge-discharge current of 50 mA/cm^2^. After 1000 cycles, the residual capacitance of NF/NiCo_2_O_4_/Ni(OH)_2_ is 96.5%, which is much higher than that of NF/Co_3_O_4_/Ni(OH)_2_ (74.4%).

It is found that the NF/NiCo_2_O_4_/Ni(OH)_2_ has much better capacitive performances than NF/Co_3_O_4_/Ni(OH)_2_. The more electrochemically active nature of the NiCo_2_O_4_ nanowire than Co_3_O_4_ plays a role, however the much enhanced mass-loading of Ni(OH)_2_ shell on the NiCo_2_O_4_ core than Co_3_O_4_ contribute more to their capacitances. In order to find out the reasons for the loading increase of the Ni(OH)_2_ shells on the NiCo_2_O_4_ core, the microstructures of the cores, especially the surface area, need to be examined, because the cores instead of bare nickel foams now serve as the effective substrates for the Ni(OH)_2_ growth.

**Figure 7:**
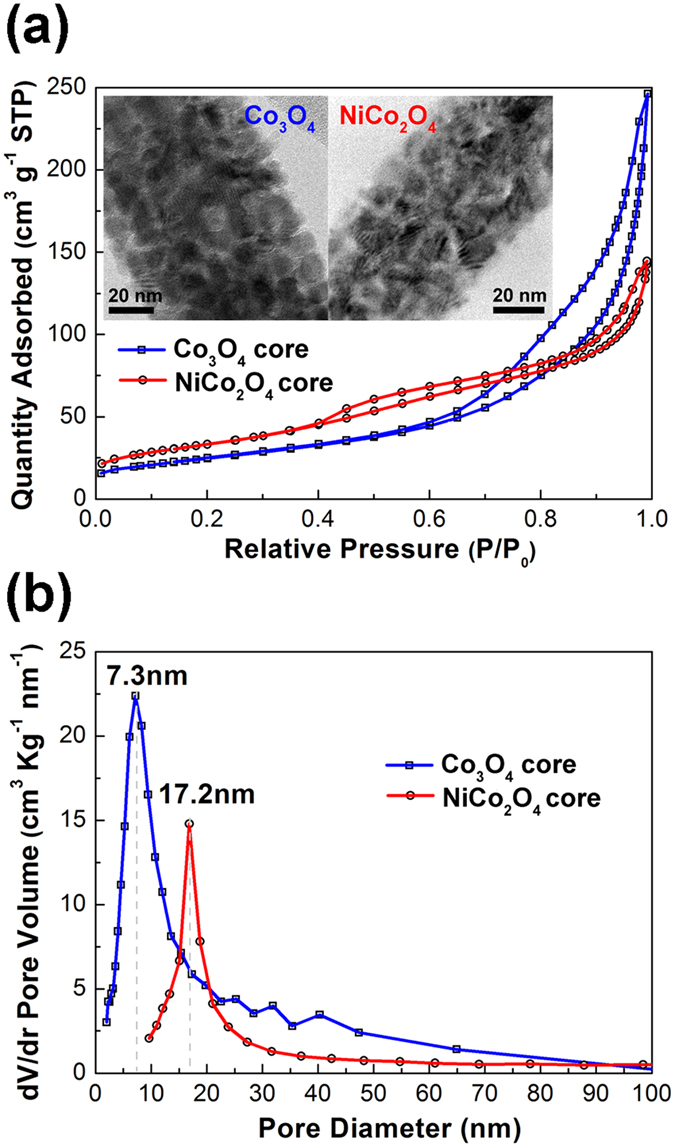
(**a**) Nitrogen adsorption-desorption isotherms with TEM images in the insets and (**b**) pore size distribution curves of the Co_3_O_4_ and NiCo_2_O_4_ nanowire cores.

The analysis of the surfaces and pores of the Co_3_O_4_ and NiCo_2_O_4_ cores is performed through N_2_ adsorption-desorption isotherm together with TEM images as shown in Fig. 7. The mesoporous nature of the nanowire cores are revealed by a type-IV adsorption-desorption isotherm for both Co_3_O_4_ and NiCo_2_O_4_ in Fig. 7a^56^. The hysteresis loop for the Co_3_O_4_ can be further classified to type H3, which indicates slit-like pores. The hysteresis loop for the NiCo_2_O_4_ belongs to type H4, indicating slit-like pores with larger pore sizes^57^. In addition, the hysteresis loop of the Co_3_O_4_ core initiates at a larger relative pressure than NiCo_2_O_4_, suggesting a relative smaller pore size in the Co_3_O_4_ core^57^. In Fig. 7b, the Barret-Joyner-Halenda (BJH) pore size distribution curves give a pore size of 7.3 nm for the Co_3_O_4_ and 17.2 nm for the NiCo_2_O_4_. In the TEM images in Fig. 7a, narrow slit-like pores are formed as the gaps between the Co_3_O_4_ or NiCo_2_O_4_ grains, and the size of pores in the NiCo_2_O_4_ is relatively larger than that in the Co_3_O_4_. Besides, the larger area enclosed by the hysteresis loop of the Co_3_O_4_ nanowire than that of the NiCo_2_O_4_ nanowire indicates its bigger pore volume and the Brunauer-Emmet-Telleer (BET) surface areas of the Co_3_O_4_ and NiCo_2_O_4_ cores are 146.6 and 115.8 m^2^ g^−1^, respectively.

Table 1(I) reveals that the total surface areas of the NF/Co_3_O_4_ and NF/NiCo_2_O_4_ electrodes (2 cm × 2 cm) are 2.83 m^2^ and 2.42 m^2^, respectively. When considering the mass-loadings of their Ni(OH)_2_ shells, it yields a specific Ni(OH)_2_ loading value of 15.8 mg/m^2^ for the NiCo_2_O_4_, which is 61% higher than that for the Co_3_O_4_ (9.7 mg/m^2^). This significant enhancement of the loading capability of Ni(OH)_2_ on the NiCo_2_O_4_ in contrast to Co_3_O_4_ implies a notable change in the growth rate of Ni(OH)_2_ on Co_3_O_4_ by replacing 1/3 cobalt with nickel at the same deposition duration.

Generally, growth rate (*r*) follows a kinetic law written as^58^,
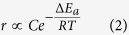
where *R* and *C* are constants, and Δ*E*_*a*_ is the chemical affinity, *i.e.* the system energy change before and after growth. In this case, Δ*E*_*a*_ is defined as the adsorption energy and expressed as:
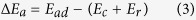
Here, *E*_*ad*_ is the total energy per cluster of Ni(OH)_2_ unit on respective nanowire core system, *E*_*c*_ is the total energy of clean systems and *E*_*r*_ is the total energy of per cluster of Ni(OH)_2_.

**Figure 8:**
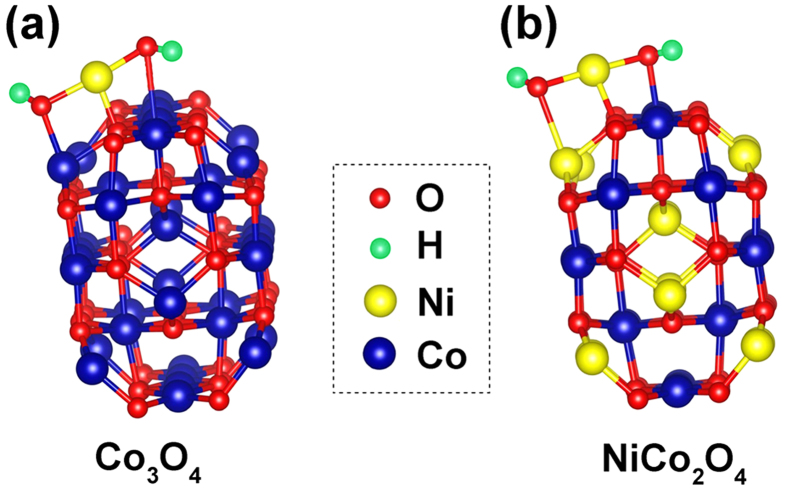
Molecular models of Ni(OH)_2_ on (**a**) Co_3_O_4_ and (**b**) NiCo_2_O_4_ for adsorption energy simulation.

To reveal this variation of chemical affinity between the cores and Ni(OH)_2_ shell, the adsorption energy of Ni(OH)_2_ unit on the core surfaces are calculated using density functional theory (DFT) with the generalized gradient approximation of Perdew and Zunger and Ion cores modeled with projector augmented wave (PAW) potentials as implemented in the VASP^59^,^60^,^61^. All calculations were performed with an energy cutoff of 500 eV, which had been tested for total energy convergence. For unit-cell calculations, a dense Monkhost-Pack grid of 8 × 8 × 8 k-ponits sampling was used and reduced to 2 × 2 × 1 k-ponits samples on nanowire or surface calculations. The convergence for energy was chosen as 10^−5^ eV between two ionic steps, and the maximum force allowed on each atom is 0.02 eVÅ^−1^. The cluster of Ni(OH)_2_ sourced from Ni(OH)_2_ crystal with a Trigonal *P-3M1* structure was put in a cubic box with the lattice constant of 15 Å, the relative ground state energy was used to as reference state energy. Here, Co_3_O_4_ nanowire is considered as the prototype, which has 36 Co and 48 O atoms in its primitive unit cell. The ideal nanowire is cut initially from optimized bulk Co_3_O_4_ crystal and, subsequently, all atoms are fully optimized. Upon relaxation, the structure of ideal clean Co_3_O_4_ nanowire reconstructed and which was adopted for adsorption calculations shown in Fig. 8a. Using Ni to replace tetrahedron Co in Co_3_O_4_ nanowire forms NiCo_2_O_4_ nanowire containing 24 Co, 12 Ni and 48 O atoms as shown in Fig. 8b.

After simulation, theoretical adsorption energy (Δ*E*_*a*_) values of −3.10 eV for Ni(OH)_2_ on NiCo_2_O_4_ and −2.17 eV for Co_3_O_4_ are calculated. The negative values reveal decreased total energies for both cases after deposition. But the lower Δ*E*_*a*_ of the NiCo_2_O_4_/Ni(OH)_2_ core/shell indicates a more favorable growth of Ni(OH)_2_ on the NiCo_2_O_4_ than Co_3_O_4_, which is well supported by the experimental observation of a much higher mass-loading of Ni(OH)_2_ on the NiCo_2_O_4_ than Co_3_O_4_ (see Table 1(I)). As a result, the capacitive performances of the core/shell electrodes are going to be affected by their respective cores and subsequent Ni(OH)_2_ shells. Additionally, the active material’s parting from electrodes during cycling is one of the major concerns for the capacitance loss in literature. In our case, the calculated smaller adsorption energy between Ni(OH)_2_ and NiCo_2_O_4_ than Co_3_O_4_ also indicates a stronger connection between Ni(OH)_2_ and NiCo_2_O_4_, which is responsible to its better cycling ability as shown in Fig. 6d.

**Figure 9:**
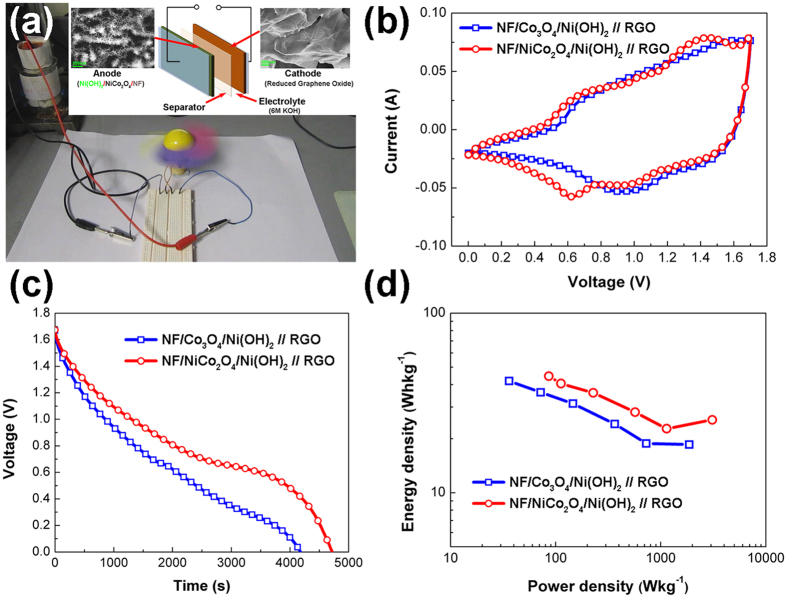
(**a**) Photograph of a working mini fan powered by the supercapacitor cell with an inset showing schematic illustration of the device structure (**b**) CV curves, (**c**) Galvanostatic discharge curves at a current density of 2.5 mA/cm^2^ and (**d**) Ragone plots for the asymmetric supercapacitors consisting of NiCo_2_O_4_/Ni(OH)_2_ and Co_3_O_4_/Ni(OH)_2_ core/shell structures and RGOs as two electrodes, respectively.

The NF/NiCo_2_O_4_/Ni(OH)_2_ electrode is applied as the anode in an asymmetric supercapacitor cell that is assembled with a NF/reduced graphene oxide (RGO) as the cathode and a 6 M KOH aqueous solution as the electrolyte (see the inset of Fig. 9a). A practical application case of this supercapacitor cell as the power supply for a mini fan is manifested in Fig. 9a.

The CV curves in Fig. 9b show great electrochemical activities of the both cell over a large potential range from 0 to 1.7 V in the aqueous electrolyte at a scan rate of 5 mV/s. But the cell consisting of NF/NiCo_2_O_4_/Ni(OH)_2_ electrode presents an extra pair of peaks around 0.6 V and 13.5 V, which could be attributed to the more active NiCo_2_O_4_ nanowire core as revealed in the half cell test (Fig. 6). Galvanostatic discharge curves shown in Fig. 9c give rise to areal specific capacitance values of 6.5 Fcm^−2^ and 7.5 Fcm^−2^ at a discharge current density of 2.5 mAcm^−2^, respectively. Additional current density dependent discharge curves are presented in Fig. S4. In the Ragone plot in Fig. 9d, the maximum energy density of the NiCo_2_O_4_ nanowire based cell is found over 44.5 Whkg^−1^ at a power density of 86.3 Wkg^−1^. Even at a high power density of 3095.3 Wkg^−1^, its energy density can reach a value of 25.5 Whkg^−1^. The Ragone plot of the Co_3_O_4_ based cell reveals an energy density 41.9 Whkg^−1^ at a power density of 36.1 Wkg^−1^ and at a relatively high power density of 1661.1 Wkg^−1^, its energy density is decreased to 18.4 Whkg^−1^. Hence, comparing with the Co_3_O_4_ nanowire based cell, the NiCo_2_O_4_ nanowire based one has overwhelming advantages in device performances and application potentials.

## Conclusions

Co_3_O_4_/Ni(OH)_2_ and NiCo_2_O_4_/Ni(OH)_2_ core/shell structures were successfully prepared on nickel form substrate through hydrothermal and chemical bath depositions. These core/shell electrodes were applied in a full asymmetry supercapacitor device with reduced graphene oxide as the cathode and KOH aqueous solution as the electrolyte. The great application potentials were demonstrated by the practical case and capacitive characterizations. More interestingly, comparative studies of the NiCo_2_O_4_/Ni(OH)_2_ and Co_3_O_4_/Ni(OH)_2_ core/shell electrodes revealed their distinct capacitive behaviors and different loading ability of Ni(OH)_2_ shell. And the causes were further investigated through theoretical simulation and surface analysis of the core/shell interfaces. It was found that the adsorption energy of Ni(OH)_2_ on NiCo_2_O_4_ is smaller than Co_3_O_4_, which resulted in more Ni(OH)_2_ shell loading and better cycling stability of NiCo_2_O_4_/Ni(OH)_2_ electrode. In this work, in addition to an exploration of the NiCo_2_O_4_ nanowire/Ni(OH)_2_ core/shell based electrode for supercapacitor applications, a comprehensive understanding of the core materials and their impacts on the core/shell structures and finally the performances of the whole cell was established. It is of great practical values to analyze various core/shell structures and develop better electrode candidates for supercapacitors.

## Methods

### Nanowires synthesis

The spinel NiCo_2_O_4_ nanowires were synthesized by a facile hydrothermal method. 5 mmol of a mixture of NiSO_4_·6H_2_O and CoSO_4_·7H_2_O (Ni:Co = 1:2), 10 mmol of NH_4_F and 25 mmol of urea were dissolved in 25 mL of de-ionized water under constant stirring. Commercially available analytical graded precursor chemicals were used without further purification. After 10 minutes of stirring, a transparent homogeneous solution was obtained and transferred into Teflon-lined stainless steel autoclaves. Clean nickel foams (2 cm × 2 cm) were subsequently immersed into the above aqueous solution. The sealed autoclave was then heated at 120 °C for 5 hours, and then naturally cooled down to room temperature. The NiCo_2_O_4_ nanowire coated nickel foam was ultrasonicated in de-ionized water for 1 minute, followed by drying at 120 °C and calcination at 250 °C in air. The Co_3_O_4_ nanowires were synthesized and treated similarly, except that no Ni precursor was used.

### Core/Shell nanowires synthesis

To grow Ni(OH)_2_ shell, a precursor was prepared by mixing 40 mL of 1 M NiSO_4_·6H_2_O, 30 mL of 0.25 M K_2_S_2_O_4_, 10 mL of aqueous ammonia (24% NH_3·_H_2_O) and 20 mL of de-ionized water in a 250 mL Pyrex beaker at room temperature. The NiCo_2_O_4_ or Co_3_O_4_ nanowire coated Ni foam (NF/NiCo_2_O_4_ or NF/Co_3_O_4_) was immersed into the precursor. After 15 minutes, the Ni(OH)_2_ coated NF/NiCo_2_O_4_ or NF/Co_3_O_4_, *i.e.* NF/NiCo_2_O_4_/Ni(OH)_2_ or NF/Co_3_O_4_/Ni(OH)_2_ electrode was subjected to a high speed rotation rinsing at 500 rpm for 3 minutes and then dried at 120 °C in air.

### Cathode electrode fabrication

A mixture paste of 90 wt% reduced graphene oxide (Graphene Supermarket) and 10 wt% PTFE was spread onto a 2 cm × 2 cm Ni foam to form the RGO-based cathode. Then the electrode was dried at 70 °C in air for 2 hours, pressed at 8 MPa, and then kept at 120 °C in air for 12 hours.

### Assembly of the asymmetric supercapacitor

An asymmetric supercapacitor was fabricated with an integrated NF/NiCo_2_O_4_/Ni(OH)_2_ or NF/Co_3_O_4_/Ni(OH)_2_ anode, an RGO/NF cathode and 6 M KOH electrolyte.

### Material and device characterizations

The morphology and microstructure of the synthesized core/shell electrodes were characterized by scanning electron microscopy (SEM, Zeiss) and transmission electron microscopy (TEM, JEOL 2010) with energy dispersive X-ray (EDS) analyzer. The structure was measured by X-ray diffraction (XRD) (BRUKER D8 ADVANCE), selected area electron diffraction (SAED) and high resolution TEM. The surface area and porosity of nanowire cores were evaluated by Brunauer–Emmett–Teller (BET, Micromeritics ASAP 2020) N_2_ adsorption–desorption measurements. Electrochemical measurements of single electrodes in a half cell were carried out in a three-electrode arrangement with the prepared electrode as working electrode, a platinum plate as counter electrode and a saturated calomel electrode (SCE) as reference electrode in 6 M KOH aqueous electrolyte. Cyclic voltammetry (CV) and galvanostatic charge/discharge test of respective single electrodes and full cells were evaluated by Solartron Electrochemical System SI 1287.

## Additional Information

**How to cite this article**: Yin, X. *et al*. Chemical insights into the roles of nanowire cores on the growth and supercapacitor performances of Ni-Co-O/Ni(OH)_2_ core/shell electrodes. *Sci. Rep.* **6**, 21566; doi: 10.1038/srep21566 (2016).

## Supplementary Material

Supplementary Information

## Figures and Tables

**Table 1:** (I): The values of loading of core, loading of shell, loading of core/shell, surface area of NF/core (SA-C), and simulated adsorption energy between core and shell (Δ*E*_*a*_) for the NF/Co_3_O_4_/Ni(OH)_2_ and NF/NiCo_2_O_4_/Ni(OH)_2_ electrodes.

**(I)**						
Electrodes	Loading of core (mg)	Loading of shell (mg)	Loading of core/shell (mg)	SA-C (m^2^)	Δ*E*_*a*_ (eV)	
NF/Co_3_O_4_/Ni(OH)_2_	19.3	28.3	47.6	2.83	−2.17	
NF/NiCo_2_O_4_/Ni(OH)_2_	20.9	37.7	58.6	2.42	−3.10	
**(II)**						
**Electrodes**	**Specific capacitance of core**			**Specific capacitance of core/shell**		
	G (F/g)	V (F/cm^3^)	A (F/cm^2^)	G (F/g)	V (F/cm^3^)	A (F/cm^2^)
NF/Co_3_O_4_/Ni(OH)_2_	865	21.0	4.2	1330	79.1	15.8
NF/NiCo_2_O_4_/Ni(OH)_2_	1241	32.5	6.5	2079	152.5	30.5

(II): Specific capacitance of core and specific capacitance of core/shell at a current density of 2.5 mA/cm^2^ are listed for the NF/Co_3_O_4_/Ni(OH)_2_ and NF/NiCo_2_O_4_/Ni(OH)_2_ electrodes, respectively. The gravimetric (G), volumetric (V) and areal (A) specific capacitance values are all presented.

## References

[b1] Wang G., Zhang L. & Zhang J. A review of electrode materials for electrochemical supercapacitors. Chem. Soc. Rev. 41, 797–828 (2012). 21779609 10.1039/c1cs15060j

[b2] Miller J. R. & Simon P. Electrochemical capacitors for energy management. Science 321, 651–652 (2008). 18669852 10.1126/science.1158736

[b3] Kötz R. & Carlen M. Principles and applications of electrochemical capacitors. Electrochim. Acta 45, 2483–2498 (2000).

[b4] Simon P. & Gogotsi Y. Materials for electrochemical capacitors. Nat. Mater. 7, 845–854 (2008). 18956000 10.1038/nmat2297

[b5] Conway B. E. Transition from “supercapacitor” to “battery” behavior in electrochemical energy storage. J. Electrochem. Soc. 138, 1539–1548 (1991).

[b6] Yu A., Chabot V. & Zhang J. Electrochemical supercapacitors for energy storage and delivery: fundamentals and applications. (CRC Press, Taylor & Francis Group, London 2013).

[b7] Conway B. E. Electrochemical Supercapacitor: Scientific Fundamentals and Technological Applications. (Kluwer Academic/Plenum, New York, 1999).

[b8] Zhang L. L. & Zhao X. Carbon-based materials as supercapacitor electrodes. Chem. Soc. Rev. 38, 2520–2531 (2009). 19690733 10.1039/b813846j

[b9] Béguin F. & Frackowiak E. Carbons for electrochemical energy storage and conversion systems. (CRC Press, Taylor & Francis Group, London, 2009).

[b10] Zhang Y. . Progress of electrochemical capacitor electrode materials: A review. Int. J. Hydrogen Energy 34, 4889–4899 (2009).

[b11] Bose S. . Carbon-based nanostructured materials and their composites as supercapacitor electrodes. J. Mater. Chem. 22, 767–784 (2012).

[b12] Jiang J. . Recent advances in metal oxide-based electrode architecture design for electrochemical energy storage. Adv. Mater. 24, 5166–5180 (2012). 22912066 10.1002/adma.201202146

[b13] Lokhande C., Dubal D. & Joo O.-S. Metal oxide thin film based supercapacitors. Curr. Appl. Phys. 11, 255–270 (2011).

[b14] Qu Q. . Electrochemical performance of MnO_2_ nanorods in neutral aqueous electrolytes as a cathode for asymmetric supercapacitors. J. Phys. Chem. C 113, 14020–14027 (2009).

[b15] Hu C.-C., Chang K.-H., Lin M.-C. & Wu Y.-T. Design and tailoring of the nanotubular arrayed architecture of hydrous RuO_2_ for next generation supercapacitors. Nano Lett. 6, 2690–2695 (2006). 17163689 10.1021/nl061576a

[b16] Rakhi R., Chen W., Cha D. & Alshareef H. Substrate dependent self-organization of mesoporous cobalt oxide nanowires with remarkable pseudocapacitance. Nano Lett. 12, 2559–2567 (2012). 22494065 10.1021/nl300779a

[b17] Zhao T., Jiang H. & Ma J. Surfactant-assisted electrochemical deposition of α-cobalt hydroxide for supercapacitors. J. Power Sources 196, 860–864 (2011).

[b18] Salunkhe R. R., Jang K., Lee S.-w. & Ahn H. Aligned nickel-cobalt hydroxide nanorod arrays for electrochemical pseudocapacitor applications. RSC Adv. 2, 3190–3193 (2012).

[b19] Prasad K. R. & Miura N. Electrochemically deposited nanowhiskers of nickel oxide as a high-power pseudocapacitive electrode. Appl. Phys. Lett. 85, 4199–4201 (2004).

[b20] Li H. . Amorphous nickel hydroxide nanospheres with ultrahigh capacitance and energy density as electrochemical pseudocapacitor materials. Nat. Commun. 4, 1894 (2013). 23695688 10.1038/ncomms2932 PMC3674274

[b21] Ni J., Zhao Y., Li L. & Mai L. Ultrathin MoO_2_ nanosheets for superior lithium storage. Nano Energy 11, 129–135 (2015).

[b22] Xia H., Meng Y. S., Li X., Yuan G. & Cui C. Porous manganese oxide generated from lithiation/delithiation with improved electrochemical oxidation for supercapacitors. J. Mater. Chem. 21, 15521–15526 (2011).

[b23] Xia H. . Hierarchical heterostructures of Ag nanoparticles decorated MnO_2_ nanowires as promising electrodes for supercapacitors. J. Mater. Chem. A 3, 1216–1221 (2015).

[b24] Wu M.-S. & Huang K.-C. Fabrication of nickel hydroxide electrodes with open-ended hexagonal nanotube arrays for high capacitance supercapacitors. Chem. Commun. 47, 12122–12124 (2011). 10.1039/c1cc14999g 21998822

[b25] Yan J. . Advanced asymmetric supercapacitors based on Ni(OH)_2_/graphene and porous graphene electrodes with high energy density. Adv. Funct. Mater. 22, 2632–2641 (2012).

[b26] Gogotsi Y. & Simon P. True performance metrics in electrochemical energy storage. Science 334, 917–918 (2011). 22096182 10.1126/science.1213003

[b27] Yang G.-W., Xu C.-L. & Li H.-L. Electrodeposited nickel hydroxide on nickel foam with ultrahigh capacitance. Chem. Commun. 6537–6539; doi: 10.1039/B815647F (2008). 19057771

[b28] Tao Y. . Towards ultrahigh volumetric capacitance: graphene derived highly dense but porous carbons for supercapacitors. Sci. Rep. 3, 2975 (2013). 24131954 10.1038/srep02975 PMC3797987

[b29] Alhebshi N. A., Rakhi R. B. & Alshareef H. N. Conformal coating of Ni(OH)_2_ nanoflakes on carbon fibers by chemical bath deposition for efficient supercapacitor electrodes. J. Mater. Chem. A 1, 14897 (2013).

[b30] Tang Z., Tang C. h. & Gong H. A high energy density asymmetric supercapacitor from nano-architectured Ni(OH)_2_/Carbon nanotube electrodes. Adv. Funct. Mater. 22, 1272–1278 (2012).

[b31] Dubal D. P., Gund G. S., Lokhande C. D. & Holze R. Decoration of spongelike Ni(OH)_2_ nanoparticles onto MWCNTs using an easily manipulated chemical protocol for supercapacitors. ACS Appl. Mater. Interfaces 5, 2446–2454 (2013). 23469934 10.1021/am3026486

[b32] Wu Z. . Electrostatic induced stretch growth of homogeneous β-Ni(OH)_2_ on graphene with enhanced high-rate cycling for supercapacitors. Sci. Rep. 4, 3669 (2014). 24413283 10.1038/srep03669 PMC3888982

[b33] Ni J. . Strongly coupled Bi_2_S_3_@CNT hybrids for robust lithium storage. Adv. Energy Mater. 4, 1400798 (2014).

[b34] Xia H. . Facile synthesis of hematite quantum-dot/functionalized graphene-sheet composites as advanced anode materials for asymmetric supercapacitors. Adv. Funct. Mater. 25, 627–635 (2015).

[b35] Yi H., Chen X., Wang H. & Wang X. Hierarchical TiN@Ni(OH)_2_ core/shell nanowire arrays for supercapacitor application. Electrochim. Acta 116, 372–378 (2014).

[b36] Zhang L. . Enhanced energy storage of a UV-irradiated three-dimensional nanostructured TiO_2_–Ni(OH)_2_ composite film and its electrochemical discharge in the dark. J. Electroanal. Chem. 683, 55–61 (2012).

[b37] Tian W. . Ni(OH)_2_ nanosheet@Fe_2_O_3_ nanowire hybrid composite arrays for high-performance supercapacitor electrodes. Nano Energy 2, 754–763 (2013).

[b38] Zhou W. . One-step synthesis of Ni_3_S_2_ nanorod@Ni(OH)_2_ nanosheet core–shell nanostructures on a three-dimensional graphene network for high-performance supercapacitors. Energy Environ. Sci. 6, 2216–2221 (2013).

[b39] Zhu J. . Hydrogenated CoO_x_ nanowire@Ni(OH)_2_ nanosheet core-shell nanostructures for high-performance asymmetric supercapacitors. Nanoscale 6, 6772–6781 (2014). 24828233 10.1039/c4nr00771a

[b40] Huang L. . Hybrid composite Ni(OH)_2_@NiCo_2_O_4_ grown on carbon fiber paper for high-performance supercapacitors. ACS Appl. Mater. Interfaces 5, 11159–11162 (2013). 24116974 10.1021/am403367u

[b41] Tang C.-H., Yin X. & Gong H. Superior performance asymmetric supercapacitors based on a directly grown commercial mass 3D Co_3_O_4_ @Ni(OH)_2_ core–shell electrode. ACS Appl. Mater. Interfaces 5, 10574–10582 (2013). 24090480 10.1021/am402436q

[b42] Cai D. . Three-dimensional Co_3_O_4_@NiMoO_4_ core/shell nanowire arrays on Ni foam for electrochemical energy storage. ACS Appl. Mater. Interfaces 6, 5050–5055 (2014). 24598433 10.1021/am500060m

[b43] Han L., Tang P. & Zhang L. Hierarchical Co_3_O_4_@PPy@MnO_2_ core–shell–shell nanowire arrays for enhanced electrochemical energy storage. Nano Energy 7, 42–51 (2014).

[b44] Hong W. . Controllable synthesis of CoAl LDH@Ni(OH)_2_ nanosheet arrays as binder-free electrode for supercapacitor applications. J. Alloy. Compound. 608, 297–303 (2014).

[b45] Huang L. . Nickel-cobalt hydroxide nanosheets coated on NiCo_2_O_4_ nanowires grown on carbon fiber paper for high-performance pseudocapacitors. Nano Lett. 13, 3135–3139 (2013). 23755979 10.1021/nl401086t

[b46] Liu J. . Co_3_O_4_ Nanowire@MnO_2_ ultrathin nanosheet core/shell arrays: a new class of high-performance pseudocapacitive materials. Adv. Mater. 23, 2076–2081 (2011). 21413085 10.1002/adma.201100058

[b47] Liu X. . Hierarchical NiCo_2_O_4_@NiCo_2_O_4_ core/shell nanoflake arrays as high-performance supercapacitor materials. ACS Appl. Mater. Interfaces 5, 8790–8795 (2013). 23937272 10.1021/am402681m

[b48] Lu Z. . Hierarchical Co_3_O_4_@Ni-Co-O supercapacitor electrodes with ultrahigh specific capacitance per area. Nano Res. 5, 369–378 (2012).

[b49] Ning F. . Co_3_O_4_@layered double hydroxide core/shell hierarchical nanowire arrays for enhanced supercapacitance performance. Nano Energy 7, 134–142 (2014).

[b50] Zhang H. . Hierarchical Mo-decorated Co_3_O_4_ nanowire arrays on Ni foam substrates for advanced electrochemical capacitors. J. Mater. Chem. A 1, 8593 (2013).

[b51] Zhou C., Zhang Y., Li Y. & Liu J. Construction of high-capacitance 3D CoO@polypyrrole nanowire array electrode for aqueous asymmetric supercapacitor. Nano Lett. 13, 2078–2085 (2013). 23570565 10.1021/nl400378j

[b52] Yu L., Zhang G., Yuan C. & Lou X. W. Hierarchical NiCo_2_O_4_@MnO_2_ core-shell heterostructured nanowire arrays on Ni foam as high-performance supercapacitor electrodes. Chem. Commun. 49, 137–139 (2013). 10.1039/c2cc37117k 23169236

[b53] Zhang G. . Nanoforest of hierarchical Co_3_O_4_@NiCo_2_O_4_ nanowire arrays for high-performance supercapacitors. Nano Energy 2, 586–594 (2013).

[b54] Shao M. . Core–shell layered double hydroxide microspheres with tunable interior architecture for supercapacitors. Chem. Mater. 24, 1192–1197 (2012).

[b55] Xu K. . Design and synthesis of 3D interconnected mesoporous NiCo_2_O_4_@Co_x_Ni_1−x_(OH)_2_ core–shell nanosheet arrays with large areal capacitance and high rate performance for supercapacitors. J. Mater. Chem. A 2, 10090 (2014).

[b56] Lowell S. Characterization of porous solids and powders: surface area, pore size and density. (Springer Science & Business Media, Norwell, 2004).

[b57] Rouquerol J., Rouquerol F., Llewellyn P., Maurin G. & Sing K. S. Adsorption by powders and porous solids: principles, methodology and applications. (Academic press, Elsever, Kidlington, 2013).

[b58] Teng H. H., Dove P. M. & De Yoreo J. J. Kinetics of calcite growth: surface processes and relationships to macroscopic rate laws. Geochim. Cosmochim. Acta 64, 2255–2266 (2000).

[b59] Kresse G. & Joubert D. From ultrasoft pseudopotentials to the projector augmented-wave method. Phys. Rev. B 59, 1758 (1999).

[b60] Kresse G. & Hafner J. Ab initio molecular dynamics for open-shell transition metals. Phys. Rev. B 48, 13115 (1993). 10.1103/physrevb.48.13115 10007687

[b61] Kresse G. & Hafner J. Ab initio molecular-dynamics simulation of the liquid-metal–amorphous-semiconductor transition in germanium. Phys. Rev. B 49, 14251 (1994). 10.1103/physrevb.49.14251 10010505

