# Supplementary material for: Chemical insights into the roles of nanowire cores on the growth and supercapacitor performances of Ni-Co-O/Ni(OH)2 core/shell electrodes
Source: Sci Rep. 2016 Feb 9;6:21566. doi: 10.1038/srep21566 (PMC4746664; doi:10.1038/srep21566)
Supplement: Supplementary Information [file srep21566-s1.pdf]

Supplementary information

Chemical insights into the roles of nanowire cores on the growth and supercapacitor performances of Ni-Co-O/Ni(OH)$_2$ core/shell electrodes

Xuesong Yin[1,†], Chunhua Tang[1,†], Liuyang Zhang[1], Zhi Gen Yu[2] & Hao Gong[1,*]

[1]Department of Materials Science and Engineering, National University of Singapore, 117576, Singapore. [2]Institute of High Performance Computing, 1 Fusionopolis Way, Singapore 138632, Singapore. [†]These authors contributed equally to this work. [*]Correspondence and requests for materials should be addressed to H. G. (msegongh@nus.edu.sg)

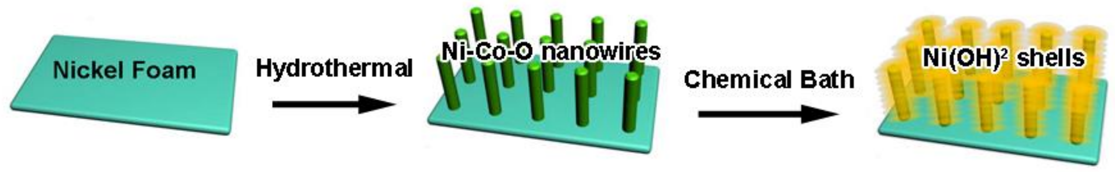

Figure S1. Schematic illustration of the fabrication process of the Ni-Co-O/Ni(OH)2 core/shell structures on nickel foam

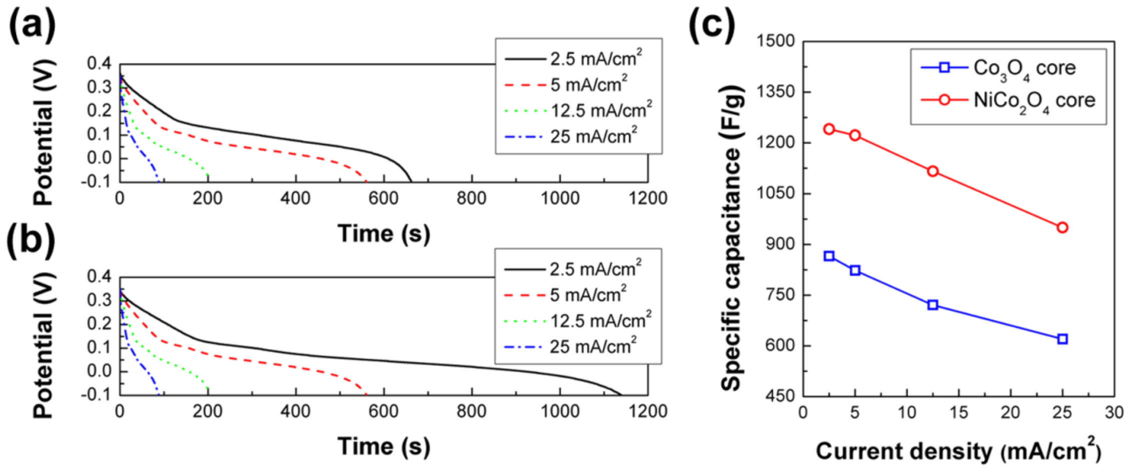

Figure S2. Galvanostatic discharge curves of half-cells with (a) NF/Co_3O_4 and (b) NF/$NiCo_2O_4$ electrodes at different current densities, and (c) plots of gravimetric specific capacitance of NF/Co_3O_4 and NF/$NiCo_2O_4$ electrodes with respect to current density.

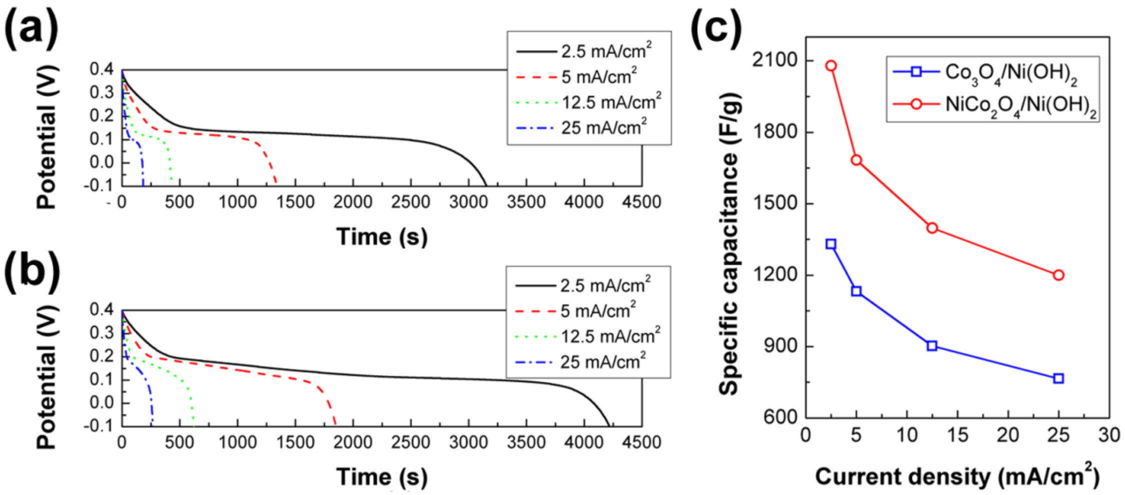

Figure S3. Galvanostatic discharge curves of half-cells with (a) NF/Co_3O_4/$Ni(OH)_2$ and (b) NF/$NiCo_2O_4$/$Ni(OH)_2$ electrodes at different current densities, and (c) plots of gravimetric specific capacitance of NF/Co_3O_4/$Ni(OH)_2$ and NF/$NiCo_2O_4$/$Ni(OH)_2$ electrodes with respect to current density.

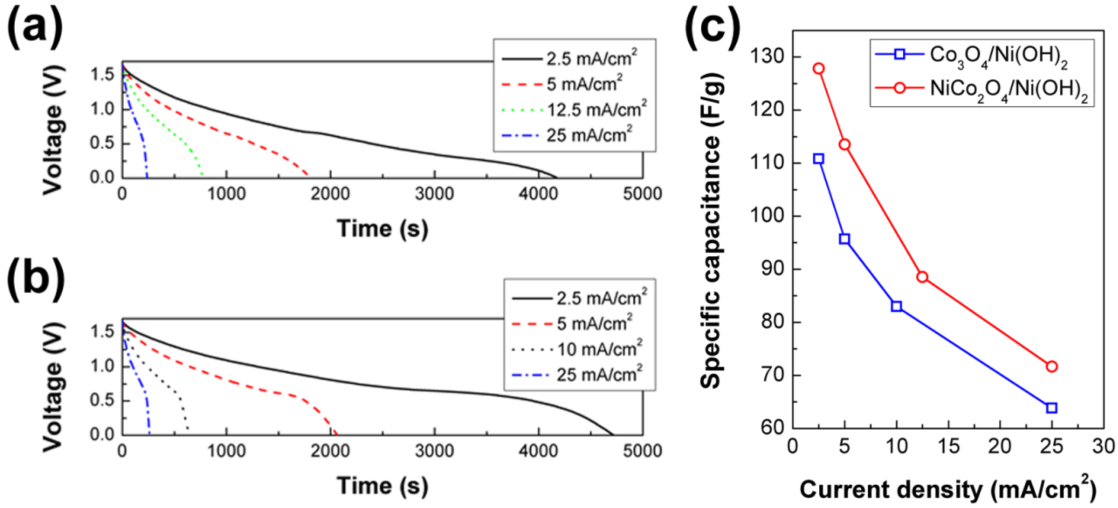

Figure S4. Galvanostatic discharge curves of full-cells with (a) NF/Co_3O_4/$Ni(OH)_2$ and (b) NF/$NiCo_2O_4$/$Ni(OH)_2$ electrodes at different current densities, and (c) plots of gravimetric specific capacitance of full-cells with NF/Co_3O_4/$Ni(OH)_2$ and NF/$NiCo_2O_4$/$Ni(OH)_2$ electrodes with respect to current density.
